# Active tuberculosis disease among people living with HIV on ART who completed tuberculosis preventive therapy at three public hospitals in Uganda

**DOI:** 10.1371/journal.pone.0313284

**Published:** 2024-11-11

**Authors:** Gaston Turinawe, Derrick Asaasira, Margret Banana Kajumba, Ivan Mugumya, Dennis Walusimbi, Florence Zawedde Tebagalika, Francis Kakooza Wasswa, Munanura Turyasiima, Susan Wendy Wandera Kayizzi, Ambrose Odwee, Khawa Namajja, Mabel Nakawooya, Paul Lwevola, Deo Nsubuga, Bruce Nabaasa, Shallon Atuhaire, Musa Dahiru, Derrick Kimuli

**Affiliations:** 1 Faculty of Science and Technology, Department of Health Sciences, Cavendish University Uganda, Kampala, Uganda; 2 Hoima Regional Referral Hospital, Hoima, Uganda; 3 Galen Health Consulting Group, Kampala, Uganda; 4 Alliance for Community Health Initiative, Kampala, Uganda; 5 Department of Standards Compliance Accreditation and Patient Protection, Ministry of Health Uganda, Nakasero, Kampala, Uganda; 6 STD/AIDS Control Program, Ministry of Health Uganda, Nakasero, Kampala, Uganda; 7 Mbarara University of Science and Technology, Mbarara, Uganda; 8 National Tuberculosis and Leprosy Program, Ministry of Health Uganda, Nakasero, Kampala, Uganda; 9 Makerere University Joint AIDS Program, United States Agency for International Development Local Partner Health Services East Central Activity, Kampala, Uganda; 10 Social & Scientific Systems., A DLH Holdings Company / United States Agency for International Development Strategic Information Technical Support Activity, Kampala, Uganda; Stellenbosch University, SOUTH AFRICA

## Abstract

Tuberculosis (TB) preventive therapy (TPT) reduces the incidence of TB among people living with the human immunodeficiency virus (PLHIV). However, despite an increase in TPT uptake, TB/HIV coinfection remains stagnant in Uganda especially in areas of increasing HIV incidence such as the Bunyoro sub-region. This study was a retrospective review records (antiretroviral therapy [ART] files) of PLHIV who were active on ART and completed TPT in 2019/2020 at three major hospitals in the Bunyoro sub-region, Uganda: Masindi General Hospital, Hoima Regional Referral Hospital, and Kiryandongo General Hospital. The sample size (987) for each facility was determined using a proportionate sampling method to ensure the study’s power and precision. Factors independently associated with acquiring TB disease post TPT were determined using modified Poisson regression analysis. An adjusted prevalence risk ratio (aPRR) with corresponding 95% confidence intervals were reported. The participants’ mean age was 38.23 (±11.70) and the majority were female (64.94%). Overall, 9.63% developed active TB disease post TPT completion. In the adjusted analysis, factors associated with active TB disease were a history of an unsuppressed viral load after TPT (aPRR 4.64 (2.85–7.56), *p*<0.001), opportunistic infections after TPT completion (aPRR 4.31 (aPRR 2.58–7.2), *p*<0.001), a history of TB active TB disease (aPRR 1.60 (1.06–2.41), *p* = 0.026), and chronic illness during or after TPT (aPRR 1.68 (1.03–2.73), *p* = 0.038). To reduce the development of TB disease post TPT thereby improving the effectiveness of TPT, ART adherence should be emphasized to resolve viral suppression and active management of chronic and opportunistic infections. Further clinical management consideration and research is needed for PLHIV who receive TPT but have a previous history of TB disease.

## Introduction

In 2022, an estimated 6.3% of all new tuberculosis (TB) cases globally were among people living with the human immunodeficiency virus (PLHIV). In some parts of southern Africa where the incidence of HIV is high, more than 50% of all TB cases were PLHIV [1]. The high occurrence of TB cases among PLHIV is partly due to their compromised immune systems [1,2]. This has created both a clinical and global health challenge in addressing TB among PLHIV. In 2022, an estimated 167,000 deaths from TB occurred among PLHIV worldwide, showing a continuous decline over the years representing 12.8% of the total 1.30 million deaths [1]. Despite the additional clinical and program management challenges posed, providing antiretroviral therapy (ART) together with anti-TB treatment increases survival rates of PLHIV with TB [3,4]. In 2023, ART and anti-TB treatment provision was estimated to have averted an estimated 6.4 million deaths between 2010 to 2022 [1].

Prevention is better than cure; therefore, TPT serves as an effective tool in reducing the incidence of TB, particularly PLHIV. TPT prevents latent TB infections from progressing to active disease, thereby curbing the spread and impact of TB [5,6]. It complements the World Health Organization’s (WHO) goal to reduce TB incidence by 50% and TB deaths by 75% by 2025 [7]. To accelerate this progress, between 2018 and 2022, an estimated 15.5 million PLHIV received TB preventive treatment [1]. In Uganda, despite the continued scale up of TPT among PLHIV, TB/HIV co-infection rates staggered at 37% according to Ministry of Health Performance data for the period of April 2023 to March 2024 [8]. Although there remains a gap with some PLHIV not yet enrolled on TPT [8], this trend highlights the urgent need to examine TB incidence among those who have completed TPT. Moreover, regions such as Bunyoro that have recently seen an increase in HIV incidence are a higher risk of increased TB-HIV co-infection [9,10]. This study examines the prevalence of active TB disease after TPT completion among PLHIV and examines contributing factors with an aim to improve the effectiveness of TB prevention strategies.

## Materials and methods

### Study design, population and setting

The study was a retrospective review of records of PLHIV who completed TPT in 2019 and 2020 and were still active on ART at the time of the study (May–June 2024) in three major public hospitals—Masindi General Hospital, Hoima Regional Referral Hospital, and Kiryandongo General Hospital—located in the Bunyoro sub-region of Western Uganda. TPT scale-up in Uganda began in 2018, making this period the earliest point of reliable data availability and providing a 4–5-year window to assess the acquisition of active TB post TPT completion.

As of recent reports, over 700,000 PLHIV of the over 1.4 million PLHIV in the country have been initiated on TPT as part of the country’s broader efforts to combat the high TB-HIV co-infection burden [6,11]. Initially, Uganda adopted the 6-month isoniazid preventive therapy (IPT) regimen for PLHIV, but the guidelines have since evolved to include shorter, more tolerable regimens like the 3-month rifapentine and isoniazid (3HP) regimen to improve adherence and reduce pill burden. The guidelines have expanded TPT eligibility beyond PLHIV to include other high-risk groups such as prisoners, miners, and healthcare workers [12,13]. Integration of TPT into routine HIV care services has been a priority, ensuring regular TB screening and counseling on adherence during ART visits, while training health workers to monitor side effects and long-term outcomes. Recent revisions emphasize adherence monitoring and pharmacovigilance, especially for newer regimens, while the guidelines now address managing latent TB infection in settings with multidrug-resistant TB. Moving forward, Uganda aims to continue expanding access to TPT, particularly in rural areas, and to explore even shorter regimens such as the 1-month 1HP regimen, all while strengthening follow-up care to monitor long-term TB reactivation risks.

Masindi General Hospital serves Masindi district and surrounding areas. The hospital offers a range of medical services, including outpatient and inpatient care, maternal and child health services, surgery, and treatment for common infectious diseases. like malaria, TB, and HIV/AIDS. Masindi General Hospital is also involved in various public health initiatives and community outreach programs to improve health outcomes in the region. Over 2,800 PLHIV are estimated to seek HIV care at the hospital.

Hoima Regional Referral Hospital is the major healthcare facility in Hoima District, also in the Western Region of Uganda. As a regional referral hospital, it serves a larger population, including several surrounding districts. The hospital is equipped with specialized departments such as surgery, pediatrics, internal medicine, obstetrics and gynecology, and orthopedics. It provides advanced diagnostic services, emergency care, and comprehensive treatment for a wide range of conditions. Hoima Regional Referral Hospital also functions as a teaching hospital, contributing to the training of medical professionals. The hospital plays a critical role in managing public health issues in the region, including TB and HIV/AIDS. Almost 6,000 PLHIV are estimated to seek HIV care at the hospital.

Kiryandongo General Hospital is in Kiryandongo District and provides essential healthcare services to the local population and also serves as a referral center for nearby health facilities. The hospital offers general medical services, including outpatient consultations, inpatient care, maternity services, and emergency care. Kiryandongo General Hospital manages other communicable diseases among services targeting TB and HIV/AIDS patients. The hospital also supports various health programs aimed at improving community health and has infrastructure to support the care of a growing population, including refugees from nearby settlements. Over 2,000 PLHIV are estimated to seek HIV care at the hospital.

### Study procedures

The sample size for each facility was determined independently using the sample size formula for proportions and applying the desired confidence level, margin of error, expected prevalence, and power for each hospital separately [14]. Each facility’s sample size ensured each facility had a sample size that maintained the overall study’s power and precision. The sample size was calculated using factoring a Z_α_-score of 1.96 for a 95% confidence, a Z_β_-score of 0.84 for the desired power (80%), an expected prevalence (*p) of* (10%) and a margin of error (E) of 5%. Based on the health facility records, the total number of PLHIV who completed TPT during 2019 were 1,550 (Masindi Hospital = 552, Hoima RR Hospital = 698 and Kiryandongo Hospital = 300). Based on the computation suggested by Cochran [14], approximately 283 patients were required per hospital. However, given the total number of patients, the study proportionally allocated a feasible total sample size among the hospitals.

Masindi Hospital (553 patients): Proportion of total patients: 552 / 1550 ≈ 0.356

  *Proportional sample size = 0.356 × 849 ≈ 302 patients*

  *Final sample size including 10% non-response rate = 332 patients.*

  Hoima RR Hospital (699 patients): 699 / 1550 ≈ 0.450

  *Proportional sample size = 0.450 × 849 ≈ 382 patients*

  *Final sample size including 10% non-response rate = 421 patients.*

  Kiryandongo Hospital (301 patients) = 301 / 1550 ≈ 0.194

  *Proportional sample size: 0.194 × 849 ≈ 169 patients*

  *Final sample size including 10% non-response rate = 182 patients.*

  ***Total Sample for the study = 987 patients***.

A list of all PLHIV who completed TPT at each hospital was abstracted using their ART Numbers. ChatGPT was used to randomly select the required number of patients from the list for each facility [15].

### Study variables and measurements

The outcome variable was the development of active TB disease after TPT completion, defined as documented active TB disease occurring between the completion of TPT and the time of the study. The independent variables were categorized into demographic characteristics and health-related/behavioral characteristics, all of which are collected during the routine service provision on the ART cards. The demographic characteristics included age, biological sex, marital status, highest level of education, having children, place of residence, and occupation. The health-related and behavioral characteristics encompassed functional status at TPT initiation, type of TPT regimen, history of tobacco use, HIV disclosure status before TPT, duration on ART, history of psychological (denial, anger, stress or bereavement) or social issues (such as non-disclosure, stigma, risky sexual behavior, discrimination or substance abuse) during counseling, appointment adherence (missed appointments), appointment keeping, side effects during ART or TPT, history of an unsuppressed viral load before TPT, history of an unsuppressed viral load after TPT, history of comorbidities before TPT, client transfer status before TPT initiation, opportunistic infections before TPT initiation, history of TB diagnosis before TPT, and history of chronic illness after TPT. Tables 1 and 2 in the results section illustrate the categorization of these variables.

**Table 1:** Comparison of demographic factors of PLHIV and active TB disease development after completing tuberculosis preventive therapy at three public hospitals in Uganda.

Variable	Categorization	Developed active TB disease after TPT completion			*p*—value
		No^¥^	Yes^¥^	Total^π^	
Age group	≤25 years	115 (91.27)	11 (8.73)	126 (12.77)	0.467
	26–44 years	529 (91.84)	47 (8.16)	576 (58.36)	
	≥45 years	248 (87.02)	37 (12.98)	285 (28.88)	
Biological sex	Female	597 (93.14)	44 (6.86)	641 (64.94)	<0.001
	Male	295 (85.26)	51 (14.74)	346 (35.06)	
Marital status	Married/cohabiting	511 (91.74)	46 (8.26)	557 (56.43)	0.019
	Never married	161 (92.53)	13 (7.47)	174 (17.63)	
	Widow(er) or Divorced	220 (85.94)	36 (14.06)	256 (25.94)	
Highest level of education	None or informal	375 (90.80)	38 (9.20)	413 (41.84)	0.547
	Primary	209 (88.94)	26 (11.06)	235 (23.81)	
	Secondary	195 (89.45)	23 (10.55)	218 (22.09)	
	Above Secondary	113 (93.39)	8 (6.61)	121 (12.26)	
Client has children	No	156 (92.31)	13 (7.69)	169 (17.12)	0.349
	Yes	736 (89.98)	82 (10.02)	818 (82.88)	
Place of residence	<5km for care hospital	430 (91.30)	41 (8.70)	471 (47.72)	0.349
	>5km from care hospital	462 (89.53)	54 (10.47)	516 (52.28)	
Occupation	Informal employment	807 (90.47)	85 (9.53)	892 (90.37)	0.754
	Formal employment	85 (89.47)	10 (10.53)	95 (9.63)	

^¥^Row percentages shown.

^π^Colum percentages shown.

^*^Statistically significant (p<0.05).

TPT–Tuberculosis preventive therapy.

Km–Kilometers.

**Table 2:** Comparison of health-related and behavioral factors of PLHIV and active TB disease development infection after completing tuberculosis preventive therapy at three public hospitals in Uganda.

Variable	Categorization	Developed active TB disease after TPT completion			*p*—value
		No^¥^	Yes^¥^	Total^π^	
Functional status at TPT initiation	Bedridden	6 (66.67)	3 (33.33)	9 (0.91)	0.015
	Not bedridden	886 (90.59)	92 (9.41)	978 (99.09)	
TPT Regimen	3HP	23 (95.83)	1 (4.17)	24 (2.43)	0.359
	INH	869 (90.24)	94 (9.76)	963 (97.57)	
History of tobacco use	No	706 (93.51)	49 (6.49)	755 (76.49)	<0.001
	Yes	186 (80.17)	46 (19.83)	232 (23.51)	
HIV disclosure status before TPT	Undisclosed	38 (97.44)	1 (2.56)	39 (3.95)	0.256
	Disclosed	837 (90.00)	93 (10.00)	930 (94.22)	
	Unknown	17 (94.44)	1 (5.56)	18 (1.82)	
Duration on ART	≤5 years	476 (87.99)	65 (12.01)	541 (54.81)	0.005
	>5 years	416 (93.27)	30 (6.73)	446 (45.19)	
History of identification of psychological issue during counselling	No	556 (92.67)	44 (7.33)	600 (60.79)	0.002
	Yes	336 (86.82)	51 (13.18)	387 (39.21)	
History of identification of social issue during counselling	No	595 (92.11)	51 (7.89)	646 (65.45)	0.011
	Yes	297 (87.10)	44 (12.90)	341 (34.55)	
Missed at least one ART facility appointment 6 months before TPT	No	668 (91.76)	60 (8.24)	728 (73.76)	0.013
	Yes	224 (86.49)	35 (13.51)	259 (26.24)	
Represented at any ART facility appointment 12 months before TPT	No	515 (92.29)	43 (7.71)	558 (56.53)	0.02
	Yes	377 (87.88)	52 (12.12)	429 (43.47)	
Represented at any ART facility appointment during TPT	No	405 (91.84)	36 (8.16)	441 (44.68)	0.162
	Yes	487 (89.19)	59 (10.81)	546 (55.32)	
Documented side effects during ART or TPT	No	637 (90.23)	69 (9.77)	706 (71.53)	0.802
	Yes	255 (90.75)	26 (9.25)	281 (28.47)	
History of an unsuppressed viral load before TPT	No	761 (91.69)	69 (8.31)	830 (84.09)	0.001
	Yes	131 (83.44)	26 (16.56)	157 (15.91)	
History of an unsuppressed viral load after TPT	No	827 (95.28)	41 (4.72)	868 (87.94_	<0.001
	Yes	65 (54.62)	54 (45.38)	119 (12.06)	
History of comorbidities before TPT	No	754 (92.29)	63 (7.71)	817 (82.78)	0.122
	Yes	138 (81.18)	32 (18.82)	170 (17.22)	
Client transferred in from another facility before TPT initiation	No	725 (90.97)	72 (9.03)	797 (80.75)	0.197
	Yes	167 (87.89)	23 (12.11)	190 (19.25)	
Diagnosed with an opportunistic infection before TPT initiation	No	713 (92.36)	59 (7.64)	772 (78.22)	<0.001
	Yes	179 (83.26)	36 (16.74)	215 (21.78)	
Diagnosed with an opportunistic infection before TPT initiation	No	808 (95.40)	39 (4.60)	847 (85.82)	<0.001
	Yes	84 (60.00)	56 (40.00)	140 (14.18)	
Previous history of TB diagnosis before TPT	No	816 (92.10)	70 (7.90)	886 (89.77)	<0.001
	Yes	76 (75.25)	25 (24.75)	101 (10.23)	
History of a chronic illness after TPT	No	817 (93.16)	60 (6.84)	877 (88.86)	<0.001
	Yes	75 (68.18)	35 (31.82)	110 (11.14)	

^¥^Row percentages shown.

^π^Colum percentages shown.

INH—Isoniazid.

3HP—isoniazid and rifapentine.

TPT–Tuberculosis preventive therapy.

ART–Antiretroviral Therapy.

### Data collection and processing

#### Statistical analysis

Data were abstracted between 10^th^ and 14^th^ June 2024 from ART client files using a pretested online questionnaire designed with data quality checks in Kobo Toolbox [16]. The questionnaire used in this study was developed and pretested using Kobo Toolbox, ensuring clarity and relevance through feedback from a small sample. Trained research assistants conducted data abstraction for the ART charts. In cases of missing data from client files, efforts were made to retrieve the information from alternative sources, such as registers and electronic medical records (EMR). Age was summarized as mean and standard deviation, and all variables were categorized and descriptively summarized before bivariate analysis. During the bivariate analysis, differences in the development of active TB disease after TPT completion were compared with categorized variables using the Chi-square or Fisher’s exact test, as appropriate. In addition, the study used the Directed Acyclic Graph (DAG) to identify possible colliding variables. These were excluded from the analysis, otherwise, variables with p-values less than 0.05 at univariate analysis were considered statistically significant and included in the multivariate analysis. At multivariable analysis, both logistic regression analysis and modified Poisson regression analysis with robust error variance were performed. However, due to the level of prevalence of the outcome and to limit over estimation, the modified Poisson regression analysis was preferred and finally presented [17]. The final model had robust error, achieved convergence and avoided mild violations of the assumptions of Poisson regression [18]. The final mode had Akaike Information Criterion (AIC) of 0.49, Bayesian Information Criterion (BIC) of -6442.78, and a Log pseudolikelihood of -224.42. This demonstrated a good balance between fitness and complexity, good performance for the number of parameters and adequacy in capturing the relationship between the variables without overfitting. The analysis was conducted in Stata version 15.1.

#### Ethical consideration

This study received internal review and clearance from the Internal Research Committee of Cavendish University Uganda. Additionally, ethical approval and clearance were sought from the Mildmay Uganda Research Ethics Committee (MUREC) under protocol MUREC-2024-418. Administrative approval was also secured from the study facilities, including Hoima Regional Referral Hospital, Masindi, and Kiryandongo General Hospitals, before conducting the study. To protect patient confidentiality, all data were anonymized, and unique identifiers were used to ensure that individual patients could not be traced. No data on personal identifiers was collected during the study processes, and all records were handled according to strict confidentiality protocols. The findings were reported following the Strengthening the Reporting of Observational Studies in Epidemiology guidelines [19], the principles expressed in the Declaration of Helsinki [20] and PLOS ONE Journal’s Clinical Studies Recommendations [21].

## Results

### Participant characteristics and the prevalence of active TB disease after TPT

The study examined records of 987 participants who had started and completed TPT during 2019 and 2020. The mean age of the participants was 38.23 (±11.70) years, with ages ranging from 2 to 78 years. Most of the participants were female (64.94%), married or cohabiting (56.43%), had informal or unknown education status (41.84%), had children (82.88%), lived more than 5 km from the hospital where they sought ART care (52.28%), and were informally employed. Additionally, the majority were not bedridden at TPT initiation (99.09%), were initiated on the TPT INH regimen (97.57%), had no history of tobacco, alcohol, or drug use (76.49%), and had been on ART for less than 5 years (54.81%). Further details of the participant characteristics are shown in Tables 1 and 2. Overall, 9.63% (95% CI: 7.86–11.64) of the participants developed active TB disease after successful completion of TPT.

### Demographic factors associated with active TB disease after TPT

Compared to their counterparts, clients who developed active TB disease after completing TPT were more likely to be aged ≥45 (12.98%), male (14.74%), widowed or divorced (14.06%), had secondary education as their highest level of education (10.55%), had children (10.02%), lived more than 5 km from the hospital providing ART care (10.47%), and had formal employment (10.53%). Statistically significant differences in the proportion of clients who developed active TB disease after TPT completion were observed only with sex (female: 6.86% vs. male: 14.74%, *p*<0.001) and marital status (married: 8.26% vs. never married: 7.47% vs. widowed/divorced: 14.06%, *p* = 0.019). Table 1 provides detailed findings of the comparison between demographic factors and the development of active TB disease after completion of TPT.

### Health-related and behavioral factors associated with the development of active TB disease after TPT

Compared to their counterparts, a higher proportion of clients who got active TB disease after completing TPT were those who were bedridden at TPT initiation (33.33%), on INH (9.76%), had a history of tobacco use (19.83%), had disclosed their HIV status (10.00%), were on ART for less than 5 years (12.01%), had a history of psychological issues (13.18%) or social issues (12.90%), missed an ART appointment in the 6 months preceding TPT start (13.51%), had been represented for an ART appointment in the 12 months preceding TPT start (12.12%) or during TPT (10.81%), had no documented side effects on TPT (9.77%), had a history of an unsuppressed viral load before (16.56%) or after TPT (45.38%), had documented comorbidities before TPT (18.82%), had transferred from another facility before TPT initiation (12.11%), had a history of opportunistic infections before (16.74%) or after TPT (40.00%), had a previous TB diagnosis before TPT (24.75%), and had any chronic illness after TPT (31.82%).

However, statistically significant differences were observed only for the following variables: functional status (*p* = 0.015), tobacco use (*p*<0.001), ART duration (*p* = 0.005), history of psychological issues (*p* = 0.002), history of social issues (*p* = 0.011), missed appointments (*p* = 0.013), representation for appointments (*p* = 0.02), unsuppressed viral load history before (*p* = 0.001) or after TPT (*p*<0.001), opportunistic infections before (*p*<0.001) or after TPT (*p*<0.001), previous TB diagnosis (*p*<0.001), and history of other chronic illnesses after TPT (*p*<0.001). Table 2 provides detailed findings of the comparison between health-related/behavioral factors and the development of active TB disease after TPT.

### Multivariate analysis of demographic, health-related and behavioral factors of PLHIV and active TB disease development after completing Tuberculosis preventive therapy at three public hospitals in Uganda

In the unadjusted analysis, clients were more likely to develop active TB disease if they were male (PRR 2.15 (1.47–3.15), *p*<0.001), widowed/divorced (PRR 1.7 (1.13–2.57), *p* = 0.011), bedridden at TPT initiation (PRR 3.54 (1.38–9.11), *p* = 0.006), had a history of substance use (PRR 3.06 (2.1–4.44), *p*<0.001), had psychological or social issues during counseling (psychological: PRR 1.8 (1.23–2.63), *p* = 0.003; social: PRR 1.63 (1.12–2.39), *p* = 0.012). Additionally, clients were more likely to develop active TB disease if they had an unsuppressed viral load before or after TPT initiation (PRR 1.99 (1.31–3.02), *p* = 0.001), (PRR 9.61 (6.72–13.74), *p*<0.001), had a history of other opportunistic infection(s) before starting TPT (PRR PRR 2.19 (1.49–3.22), *p*<0.001), were diagnosed with any other opportunistic infection(s) after completing TPT (PRR 8.69 (6.01–12.55), *p*<0.001), had a history of TB disease before TPT (PRR 3.13 (2.08–4.71), *p*<0.001), and had a chronic illness during or after TPT (PRR 4.65 (3.22–6.71), *p*<0.001). Clients were less likely to develop TB after TPT if they had been on ART for more than 5 years before TPT (PRR 0.56 (0.37–0.85), *p* = 0.006).

In the adjusted analysis, those more likely to develop active TB disease were clients had history of an unsuppressed viral load after TPT (aPRR 4.64 (2.85–7.56), *p*<0.001), acquired any other opportunistic infection after TPT completion (aPRR 4.31 (aPRR 2.58–7.2), *p*<0.001), had a history of TB active TB disease (aPRR 1.60 (1.06–2.41), *p* = 0.026), and had a chronic illness during or after TPT (aPRR 1.68 (1.03–2.73), *p* = 0.038) were all significantly associated with a higher likelihood of acquiring active TB disease. Table 3 shows the findings for the multivariate analysis.

**Table 3:** Multivariate analysis of demographic, health-related and behavioral factors of PLHIV and the development of active TB disease after completing tuberculosis preventive therapy at three public hospitals in Uganda.

Variable	Categorization	Prevalence Risk Ratio (PRR)	P-Value	Adjusted Prevalence Risk Ratio (aPRR)	P-Value
Biological sex	Female	1		1	
	Male	2.15 (1.47–3.15)	<0.001	1.43 (0.97–2.1)	0.067
Marital status	Married	1		1	
	Never married	0.9 (0.5–1.64)	0.740	1.4 (0.81–2.44)	0.230
	Widow(er)/Divorced	1.7 (1.13–2.57)	0.011	1.33 (0.87–2.03)	0.195
Client functional status at TPT initiation	Not bedridden	1		1	
	Bedridden	3.54 (1.38–9.11)	0.009	1.82 (0.84–3.92)	0.128
Duration of client on ART before TPT initiation in years	No	1		1	
	Yes	0.56 (0.37–0.85)	0.006	0.75 (0.5–1.12)	0.154
History of identification of psychological issue during counselling	≤5 years	1		1	
	>5 years	1.8 (1.23–2.63)	0.003	0.82 (0.52–1.28)	0.379
History of identification of social issue during counselling	No	1		1	
	Yes	1.63 (1.12–2.39)	0.012	0.87 (0.58–1.3)	0.497
Did the client miss any ART clinic appointment in the 6 months before TPT?^π^	No	1			
	Yes	1.64 (1.11–2.43)	0.013		
Was the client ever represented during a clinic appointment in 12 months before starting TPT? ^π^	No	1			
	Yes	1.57 (1.07–2.31)	0.021		
History of alcohol or drug or tobacco use	No	1			
	Yes	3.06 (2.1–4.44)	<0.001	1.44 (0.97–2.14)	0.069
History of an unsuppressed viral load before TPT	No	1		1	
	Yes	1.99 (1.31–3.02)	0.001	0.75 (0.48–1.19)	0.222
History of an unsuppressed viral load after TPT	No	1		1	
	Yes	9.61 (6.72–13.74)	<0.001	4.64 (2.85–7.56)	<0.001
client diagnosed with any other opportunistic infection before initiating TPT?	No	1		1	
	Yes	2.19 (1.49–3.22)	<0.001	1.05 (0.67–1.65)	0.837
client diagnosed with any other opportunistic infection after completing TPT?	No	1		1	
	Yes	8.69 (6.01–12.55)	<0.001	4.31 (2.58–7.2)	<0.001
Was the client ever diagnosed with TB (at any point in their life) before starting TPT?	No	1		1	
	Yes	3.13 (2.08–4.71)	<0.001	1.6 (1.06–2.41)	0.026
Did the client have any chronic illness during TPT or after completing TPT?	No	1		1	
	Yes	4.65 (3.22–6.71)	<0.001	1.68 (1.03–2.73)	0.038

^π^Statistically significant variable excluded from final model due to possibility of colliding based on the Directed Acyclic Graph (DAG) considerations.

TPT–Tuberculosis preventive therapy.

ART–Antiretroviral Therapy.

Exponentiated coefficients are for risk ratios; 95% confidence intervals in brackets; PRR: Unadjusted Risk ratio; aPRR: Adjusted risk ratio. Findings from a modified Poisson regression analysis with robust error variance.

## Discussion

The findings of this study indicated that 1 in 10 PLHIV developed active TB disease after the successful completion of TPT in the three major hospitals in Bunyoro sub-region, Uganda. PLHIV who were more likely to develop active TB disease after TPT were those with history of an unsuppressed viral load post TPT, those who acquired any other opportunistic infection after TPT completion, had a history of active TB disease, and those who had chronic illness during or after TPT.

At the three main hospitals of the Bunyoro sub-region of Uganda, approximately 10% of PLHIV seeking care ART care developed active TB disease after completing TPT. This contrasts sharply with findings from studies in Ethiopia, which reported about 4% [22,23]. The difference may be attributed to the variation in follow-up durations. While the Ethiopian studies assessed clients 2–5 years after completing TPT, this study focused on clients 4–5 years post-TPT completion. The shorter follow-up period in the Ethiopian studies may primarily capture the immediate protective effects of TPT, during which the risk of TB reactivation is lower. In contrast, the longer follow-up in this study may have revealed a decline in TPT’s efficacy over time. This underscores the need to explore the potential benefits of administering repeat TPT courses, a strategy that has yet to be thoroughly investigated [24]. Nonetheless, the high prevalence of TB among PLHIV post-TPT undermines the country’s efforts towards reducing the TB 90% by 2035 [7].

The history of an unsuppressed viral load post-TPT was the strongest predictor of the development of active TB disease post-TPT, with participants being over 4 times more likely to develop active TB disease. This finding underscores the critical role of viral suppression in preventing TB among PLHIV. Unsuppressed HIV can severely compromise the immune system, facilitating the progression from latent to active TB [6,25]. Since an unsuppressed viral load is usually attributed to poor adherence [25], it is likely that poor ART adherence after TPT influenced to post-TPT active TB disease. It is therefore imperative to emphasize continuation of adherence to ART TPT and monitoring of viral load suppression to prevent active TB disease post-TPT.

The occurrence of other opportunistic infections after TPT completion was strongly associated with active TB disease. Opportunistic infections further weaken the immune system, making it difficult to fight off TB bacteria [26]. Therefore, continuous monitoring and management of opportunistic infections are essential post-TPT completion.

Participants with a history of active TB disease were 60% more likely to develop TB post-TPT. Although this recurrence may be due to residual TB bacteria or reinfection in high-burden settings [27,28], TPT contains anti-TB drugs that could as well help with this [23]. However, it is likely that the previous risk factors that led to past active TB disease if not addressed could lead to future TB episodes post TPT. Recurrent TB episodes may exacerbate the risk of drug-resistance, a challenge seen in the country [29]. Therefore, post-TPT, cautious monitoring of PLHIV with a history of TB is important. Moreover, emerging literature on post-TB lung disease points to chronic lung damage as a key biological mechanism that increase vulnerability to TB recurrence [30,31]. Consequently, this reduces the effectiveness of TPT.

While studies in the country have examined the uptake and or completion rates for TPT among PLHIV [6,11,32], this study stands out as it examined prevalence of TB after TPT. Consequently, its findings help improve TB prevention among PLHIV and tamper TPT expectations. However, as a retrospective study of patient records, it was subject to inherent design limitations such as the reliance on existing records means. Some relevant information might have been omitted or inaccurately documented, affecting the robustness of the data. Additional limitations include the inability to establish causality, limited study area and lack of control of additional confounders that could influence the development of active TB post-TPT that were not documented. Despite these limitations, the study provides valuable insights into the factors associated with the development of active TB disease among PLHIV who have completed TPT, and its limitations highlight important areas for future research and intervention.

## Conclusions

One in ten PLHIV who successfully completed TPT at three hospitals during 2019/20 developed active TB disease. Several factors were associated with post-TPT TB, underscoring the multifaceted nature of TB risk in this vulnerable population. A history of unsuppressed viral load post-TPT, occurrence of opportunistic infections after TPT, prior active TB disease, and chronic illnesses during or after TPT completion significantly increased the likelihood of developing active TB. This study highlights the need for more targeted public health strategies at both the facility and national levels to improve TPT effectiveness. To achieve this, additional approaches may be needed, including exploring the potential role of repeat TPT doses and implementing active monitoring of PLHIV sub-groups at higher risk of TB, as identified by this study. To minimize the risk of TB recurrence and drug resistance, it is essential to establish rigorous post-TPT monitoring for PLHIV with a history of TB, as chronic lung damage and unresolved risk factors from previous TB episodes could significantly undermine the effectiveness of TPT.

## Supporting information

S1 Dataset (XLSX)

S1 File Questionnaire. (PDF)
