# Supplementary material for: Active tuberculosis disease among people living with HIV on ART who completed tuberculosis preventive therapy at three public hospitals in Uganda
Source: PLoS One. 2024 Nov 11;19(11):e0313284. doi: 10.1371/journal.pone.0313284 (PMC11554154; doi:10.1371/journal.pone.0313284)
Supplement: S1 File — (PDF) [file pone.0313284.s002.pdf]

# Factors associated with increased cases of TB among PLHIV who received and completed TB Preventative Therapy (TPT) for the period 2020

**Client ART No**

______________________________

**1. Age of client in completed years at TPT initiation**

______________________________

**2. Biological sex of client**

- ◯ Male
- ◯ Female

**3. Marital status of client**

- ◯ Single (Never married)
- ◯ Married/cohabiting
- ◯ Widowed, Widower or Divorced

**4. Highest level of education**

- ◯ No formal education (or Unknown)
- ◯ Primary
- ◯ Secondary
- ◯ Above Secondary

**5. Does the client have any children?**

- ◯ No
- ◯ Yes

**6. Place of residence**

- ◯ Urban (within 5km of hospital)
- ◯ Rural (More than 5km from the hospital)

**7. Occupation of client**

- ◯ Formal employment
- ◯ Informal employment

**7 (b) Specify the category if informal employment**

- Casual laborer (Fisher folk, Trucker, Plantation Worker)
- Key population (Engaged in any kind of transactional sex)
- Industrial worker (Those working at new industries)
- Other

**8. Client functional status at TPT initiation**

- Not Bed ridden
- Bed ridden

**9. What type of TPT regimen the client was initiated on?**

- 3HP
- INH

**10. Does the client have a history of alcohol or drug or tobacco use?**

- No
- Yes

**11. Had the patient disclosed their HIV status before TPT initiation?**

- No
- Yes
- Unknown

**12. Duration of client on ART before TPT initiation in years**

____________________

**13. How many counselling sessions did the client receive before starting ART?**

____________________

**14. How many counselling sessions did the client receive before starting TPT?**

____________________

**15. Were any psychological or emotional issues identified at any point during ART or TPT counselling?**

- No
- Yes
- Unknown or undocumented

**15 (b). Were the psychological or emotional issues resolved after identification?**

- No
- Yes

**16. Were any social issues identified at any point during ART or TPT counselling?**

- No
- Yes
- Unknown or undocumented

**16 (b). Were the social issues resolved after identification?**

- No
- Yes

**17. Were any spiritual issues identified at any point during ART or TPT counselling?**

- No
- Yes
- Unknown or undocumented

**17 (b). Were all the spiritual issues identified resolved after indentification?**

- No
- Yes

**18. Did the client miss any ART clinic appointment in the 6 months before TPT?**

- No
- Yes

**19. Was the client ever represented during a clinic appointment in 12 months before starting TPT?**

- No
- Yes

**20. Was the client ever represented during a clinic appointment during or after completion of TPT?**

- No
- Yes

**21. Has the client had any documented side effects during ART or TPT uptake?**

- No
- Yes

**22. Did the client have any documented adverse events during ART or TPT uptake?**

- No
- Yes

**23. Did the client ever have an unsuppressed viral load at any time before TPT initiation?**

- No
- Yes

**24. Did the client unsuppressed viral load at any time after completing TPT?**

- No
- Yes

**25.Did the client have any history of ART regiment change before TPT initiation?**

- No
- Yes - (Once)
- Yes - (More than Once)
- Unknown

**26. Did the client have any comorbidities while on ART but before TPT?**

- No
- Yes

**27. When did the client complete TPT?**

yyyy-mm-dd

---

**28. Did the client get TB after TPT? - Diagnosed with TB any time after TPT completion**

- No
- Yes

**28(a). What type of TB did the client get?**

- Pulmonary Bacteriologically Confirmed TB
- Pulmonary Clinically Diagnosed TB
- Extra Pulmonary TB

**28(b). Client functional status at TB infection**

- Not Bed ridden6
- Bed ridden

**28(c). When did the client get TB?**

yyyy-mm-dd

---

**29. Did the patient transfer in from another facility before initiating TPT?**

- No
- Yes

**30. Was the client diagnosed with any other opportunistic infection before initiating TPT?**

- No
- Yes

**31. Was the client diagnosed with any other opportunistic infection after completing TPT?**

- No
- Yes

**32. Was the client ever diagnosed with TB (at any point in their life) before starting TPT?**

- No
- Yes

**33. Did the client take any medication apart from ART during the TPT course?**

- No
- Yes

**34. Did the client have any chronic illness during TPT or after completing TPT?**

- No
- Yes

**34(b) Specify the chronic illness**

- Diabetes
- Liver or Kidney Disease
- Cancer
- Hypertension
- Other
